# Quality of online video resources concerning patient education for neck pain: A YouTube-based quality-control study

**DOI:** 10.3389/fpubh.2022.972348

**Published:** 2022-09-21

**Authors:** Xiang Zhang, Yi Yang, Yi-Wei Shen, Ke-Rui Zhang, Li-Tai Ma, Chen Ding, Bei-Yu Wang, Yang Meng, Hao Liu

**Affiliations:** Department of Orthopedics, Orthopedic Research Institute, West China Hospital, Sichuan University, Chengdu, China

**Keywords:** neck pain, YouTube, education, content analysis, quality-control study

## Abstract

**Background:**

More than 70 percent of the world's population is tortured with neck pain more than once in their vast life, of which 50–85% recur within 1–5 years of the initial episode. With medical resources affected by the epidemic, more and more people seek health-related knowledge *via* YouTube. This article aims to assess the quality and reliability of the medical information shared on YouTube regarding neck pain.

**Methods:**

We searched on YouTube using the keyword “neck pain” to include the top 50 videos by relevance, then divided them into five and seven categories based on their content and source. Each video was quantitatively assessed using the Journal of American Medical Association (JAMA), DISCERN, Global Quality Score (GQS), Neck Pain-Specific Score (NPSS), and video power index (VPI). Spearman correlation analysis was used to evaluate the correlation between JAMA, GQS, DISCERN, NPSS and VPI. A multiple linear regression analysis was applied to identify video features affecting JAMA, GQS, DISCERN, and NPSS.

**Results:**

The videos had a mean JAMA score of 2.56 (SD = 0.43), DISCERN of 2.55 (SD = 0.44), GQS of 2.86 (SD = 0.72), and NPSS of 2.90 (SD = 2.23). Classification by video upload source, non-physician videos had the greatest share at 38%, and sorted by video content, exercise training comprised 40% of the videos. Significant differences between the uploading sources were observed for VPI (*P* = 0.012), JAMA (*P* < 0.001), DISCERN (*P* < 0.001), GQS (*P* = 0.001), and NPSS (*P* = 0.007). Spearman correlation analysis showed that JAMA, DISCERN, GQS, and NPSS significantly correlated with each other (JAMA vs. DISCERN, *p* < 0.001, JAMA vs. GQS, *p* < 0.001, JAMA vs. NPSS, *p* < 0.001, DISCERN vs. GQS, *p* < 0.001, DISCERN vs. NPSS, *p* < 0.001, GQS vs. NPSS, *p* < 0.001). Multiple linear regression analysis suggested that a higher JAMA score, DISCERN, or GQS score were closely related to a higher probability of an academic, physician, non-physician or medical upload source (*P* < 0.005), and a higher NPSS score was associated with a higher probability of an academic source (*P* = 0.001) than of an individual upload source.

**Conclusions:**

YouTube videos pertaining to neck pain contain low quality, low reliability, and incomplete information. Patients may be put at risk for health complications due to inaccurate, and incomplete information, particularly during the COVID-19 crisis. Academic groups should be committed to high-quality video production and promotion to YouTube users.

## Introduction

Neck pain imposes a substantial economic burden on patients and renders them handicapped (1–5). More than 70 percent of the world's population is tortured with neck pain more than once in their vast life (6, 7), of which 50–85% are expected to recur within 1–5 years of the initial episode (7). Neck pain occurs more frequently in females than males, peaking in middle age (6, 8, 9). Neck pain is strongly associated with multiple comorbidities, including depression, headache, joint pain, and back pain (8, 9). Moreover, neck pain also poses a huge economic burden to society, including the costs of treatment, lost productivity, and job-related problems (10).

Classifying neck pain as neurotic or non-neurotic is critical and urgent, as this information is required to guide investigation (imaging necessity) and treatment decisions (11). There are many potential diagnoses for neck pain, but common ones include myofascial pain, degenerative disc disease, and muscle spasms (12). Since the prognosis of neck pain is closely related to duration (13, 14), it is important to identify patients in time and to have them receive appropriate health education and proper management.

Unfortunately, the current COVID-19 pandemic and concomitant restrictions have contributed to a severe shortage of conventional medical care (15–18). Given the explosive growth of the Internet as a source for quick and extensive information in all areas of life, 80% of netizens have searched the Internet for health information (19), and up to 30% of orthopedic patients have searched for medical information online (20), which has greatly facilitated consultation and medical treatment during the epidemic. With more than 1 billion visitors per month, YouTube boasts one of the most popular and dominant websites for viewing and sharing videos on the Internet (21, 22). Patients can use YouTube to gain health-related medical knowledge and use publicly accessible YouTube video-based tutorials to treat their conditions, especially during the current pandemic (21, 23, 24). However, while some of the high-quality orthopedic content on YouTube is uploaded by qualified specialists, it is undeniable that the majority of videos are still uploaded by unqualified individuals, and most of the videos are not peer-reviewed, resulting in the dissemination of inaccurate, incomplete, and low-quality videos. Given the lack of control mechanisms and uneven quality of uploads, there is a considerable risk of getting misleading or insufficient information on health-related issues (25, 26).

To the best of our knowledge, there currently exist no studies evaluating the reliability and quality of YouTube videos providing medical information to patients with neck pain. Especially against the backdrop of the COVID-19 pandemic, assessing the quality of the relevant videos regarding neck pain is crucial to determining the potential applicability of YouTube tutorials. This article aims to assess the quality and reliability of the medical information shared on YouTube regarding neck pain and to identify factors associated with the overall video quality or reliability.

## Materials and methods

### Recruitment

In this cross-sectional study, we used the search item “neck pain” on YouTube (accessed on 25 May 2022) and designed to include relevant videos for patients with neck pain. Search history records were deleted before the search to reduce the impact on search results. In over 50,000 search results, the top 50 videos by relevance were recorded for evaluation, which has been widely adopted as a feasible method of video selection in the literature within orthopedics (27–29). Additionally, the purpose of this study was to simulate and analyze the results viewers come across on visiting YouTube for medical information from a researcher's perspective, rather than to assess all the videos uploaded on YouTube regarding neck pain. Most YouTube viewers will not watch more than the first two pages of search results found online (30). On this basis, above YouTube video studies within orthopedics have examined small subsets of videos (50 videos) and similar statistical analyses were conducted. Therefore, we included the first 50 results for further evaluation. All videos were about neck pain and were in English.

### Collection of video features and source

The following information was recorded during screening: (1) Title, (2) number of views, (3) number of likes, (4) number of dislikes, (5) video duration, (6) comments, (7) number of subscribers, (8) time since upload and (9) Video Power Index (VPI). The formula for calculating VPI is as follows: [(likes × 100/(likes + dislikes)) × (views/day)/100]. The index used to assess viewers engagement and video popularity has been successfully applied to previous research (28, 31, 32).

According to previous studies (27, 28, 31), the videos were categorized into six groups based on their source: (1) academic (authors were affiliated with universities or research groups or colleges), (2) physician (authors were professional physicians with no affiliations), (3) non-physician (health professionals other than licensed physician: physiotherapists, and occupational therapists), (4) medical (contents from health websites), (5) commercial and (6) individuals. The categories are as follows according to content (1) exercise training, (2) information about disease, (3) patient experience, (4) non-surgical, and (5) advertisement according to previous studies. When the videos have more than one topic, we typically evaluate and identify the topics that patients will gain the most from the video. The duration of the topic, the amount of information contained, and the comments of the viewers are all important evaluation indicators.

### Assessment of reliability and quality

The accuracy and reliability of videos were assessed with the Journal of American Medical Association (JAMA) benchmark criteria, provided by Silberg et al. (33) (Table 1). The JAMA total score is calculated by assigning 1 point for the existence of each criterion. A score of 0 indicates low video quality and accuracy, while a score of 4 indicates high video quality and accuracy. The educational value of each video was scored using a 5-point global scale [recorded as the Global Quality Score (GQS)] modified from Singh et al. (34) (Table 2). GQS score ranges from 1 to 5, with higher scores indicating better quality of education. Additionally, a modified DISCERN tool, similar to the five-point evaluation tool reviewed by Kocyigit et al. (24), was used to evaluate the reliability of the included videos. It contains five binary yes/no questions, each positive yielding 1 point, with a maximum score of 5 (Table 3).

**Table 1:** Journal of the American Medical Association benchmark criteria (33).

**Criterion**	**Description**
Authorship	Author and contributor credentials and their affiliations should be provided.
Attribution	All copyright information should be clearly listed, and references and sources for content should be stated.
Currency	The initial date of posted content and dates of subsequent updates to content should be provided.
Disclosure	Conflicts of interest, funding, sponsorship, advertising, support, and video ownership should be fully disclosed.

**Table 2:** Global Quality Score criteria (34).

**Grade**	**Description of quality**
1	Poor quality, information missing, technique misleading; unlikely to be useful for patient education
2	Generally sparse quality, some information provided but majority lacking, technique poor; limited use for patients
3	Moderate quality, important information provided but some lacking, technique mostly adequate; somewhat useful for patients
4	Good quality, majority of information provided but some information lacking, technique adequate; useful for patients because most important topics are covered
5	Excellent quality, full information provided, technique adequate; highly useful for patients.

**Table 3:** 5-point DISCERN criteria (1 point for each item; a total of 5 points) (24).

**Item**	**Criteria**
1	Are aims clear and achieved?
2	Are reliable sources of information used? (published articles cited, a specialist's opinion)
3	Is information presented balanced and unbiased?
4	Are additional sources of information listed for patient reference
5	Are areas of uncertainty addressed?

Since no assessment tool exists to comprehensively assess the educational content of neck pain, we created a new grading criteria [called the “Neck Pain Specific Score” (NPSS)] based on literature review and expert opinion (5, 10, 11, 35–39). The NPSS specifically assesses educational content on (1) patient presentations, (2) information about neck pain, (3) diagnosis and evaluation, (4) treatments, and (5) post-operative course (Table 4). A single point is assigned for the presence of each item, conferring a maximum score of 18, with higher scores indicating better education quality for neck pain.

**Table 4:** The neck pain-specific score.

**Patient presentation 2**
Describes symptoms: pain location, sensory deficits, muscle weakness, reflex abnormalities, etc.
Describes patient population: both the incidence and prevalence of neck pain increased with age and were greater among females than males, etc.
**Information about neck pain 3**
Mentions epidemiology and burden: neck pain was found to rank 21st in terms of overall burden and fourth in terms of overall disability; neck pain had an age-standardized point prevalence of 3,551/100,000 people, with a 95% uncertainty interval (UI) from 3,140 to 3,978; and an annual incidence of 807/100,000 people (95% UI 714 to 913).
Describes the potential cause: cervical facet joint disease, stenosis at the cervical intervertebral foramen, osteophyte growth at the uncovertebral joints, etc.
Mentions risk factors: psychopathology, genetics, sleep problems, smoking, obesity, low job satisfaction and poorly perceived work support, etc.
**Diagnosis and evaluation 7**
Red flags: age related factors, physical signs and symptoms, miscellaneous and neurological findings, etc.
Referring to taking a comprehensive history.
Mentions physical examination: Spurling, Neck distraction, Valsalva, Hoffmann sign, and Jackson compression, etc.
Mentions the diagnostic imaging: X-ray, CT or MRI (sometimes used to confirm or rule out a specific pathology).
Mentions the classification: neuropathic, non-neuropathic, mixed neuropathic-nociceptive
Describes surgical candidates: When neck pain is associated with progressive neurologic deficits or spinal cord compression, a surgical opinion is indicated.
Mentions the prognosis: younger age, an active coping style and optimistic outlook appear to be related to a favorable prognosis; previous episodes of neck pain, concurrent low back pain, concurrent headaches, poor health, psychological factors (such as anxiety, worry, frustration and depression) and work-related symptoms (such as low job satisfaction) appear to be related to a poor prognosis.
**Treatment 4**
Mentions exercise and integrative medicine treatments: exercise, massage, spinal manipulation, Electrotherapy, yoga, education and qigong.
Mentions medication: paracetamol or non-steroidal anti-inflammatory drugs (NSAIDs).
Mentions injections: glucocorticoid, etc.
Mentions surgery.
**Post-operative course 2**
Describes outcomes and complications.
Mentions post-operative mobilization and physiotherapy including rapid recovery.

Two independent orthopedic doctors (XZ and YY) assessed all included videos. Any discrepancies were settled by discussion with a third author for consensus (HL).

### Ethics statement

Since YouTube videos with publicly available data was used, ethics committee approval was not required.

### Statistical assessments

IBM SPSS Statistics 22.0 software was utilized to analyze the results. Continuous variables are presented as the mean ± SD. Since the parameters did not show a normal distribution, we used the Kruskal-Wallis test for the between-group comparisons, followed by the Bonferroni method for *post-hoc* tests. Spearman correlation analysis was used to evaluate the correlation between JAMA, GQS, DISCERN, NPSS, and VPI. A multiple linear regression analysis was applied to identify video features affecting JAMA, GQS, DISCERN, and NPSS. For the video quality assessment, we use the intraclass correlation coefficient (ICC) to evaluate the scores between reviewers (XZ and YY). A guideline for evaluating ICC values was applied: excellent (>0.90), good (0.75–0.90), moderate (0.50–0.75) and poor (<0.50) (40). All reported *p*-values were two-sided, and those <0.05 were considered statistically significant.

## Results

### Video characteristics

**Figure 1:**
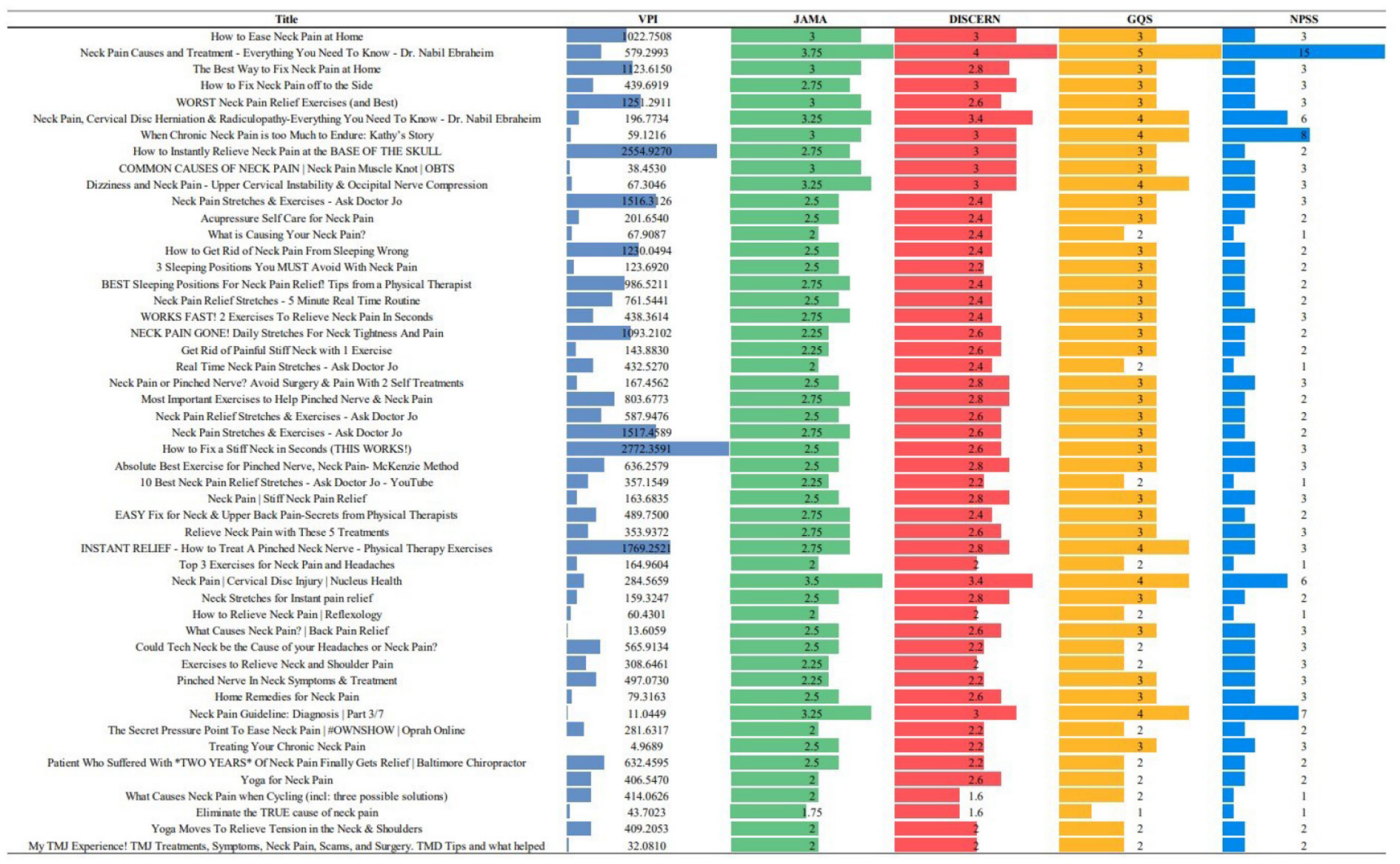
The video title, views per day, VPI, JAMA score, DISCERN, and GQS of the 50 videos are listed.

A total of 50 videos were statistically analyzed. The videos occupied a total running time of 23,197 s and an average duration of 464 s (SD = 283). They were viewed an average of 581 times per day (SD = 626) for a total of 29,054 times per day. The mean time since upload was 1,504 days (SD = 1,104) and the average likes ratio was 96.78 (SD = 2.32). The videos received an average of 0.70 (SD = 1.16) comments per day, with an average of 12.43 (SD = 19.75) likes and 0.24 (SD = 0.28) dislikes per day. The mean VPI was calculated as 566.35 (SD = 613.49) (Figure 1).

### Topics and uploading sources

**Figure 2:**
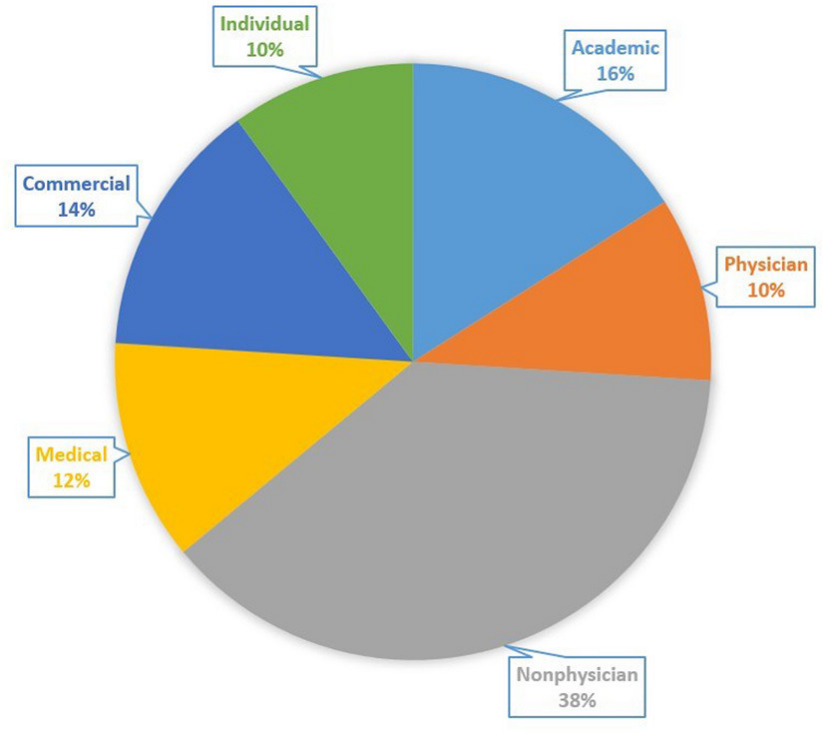
Categorical distribution of the videos based on source.

The included videos are categorized by upload sources and contents covered therein. Non-physician video sources accounted for the largest share (19/50, 38%), followed by academic sources (8/50, 16%), commercial sources (7/50, 14%), medical sources (6/50, 12%), individual sources (5/50, 10%) and physicians (5/50, 10%) (Figure 2).

**Figure 3:**
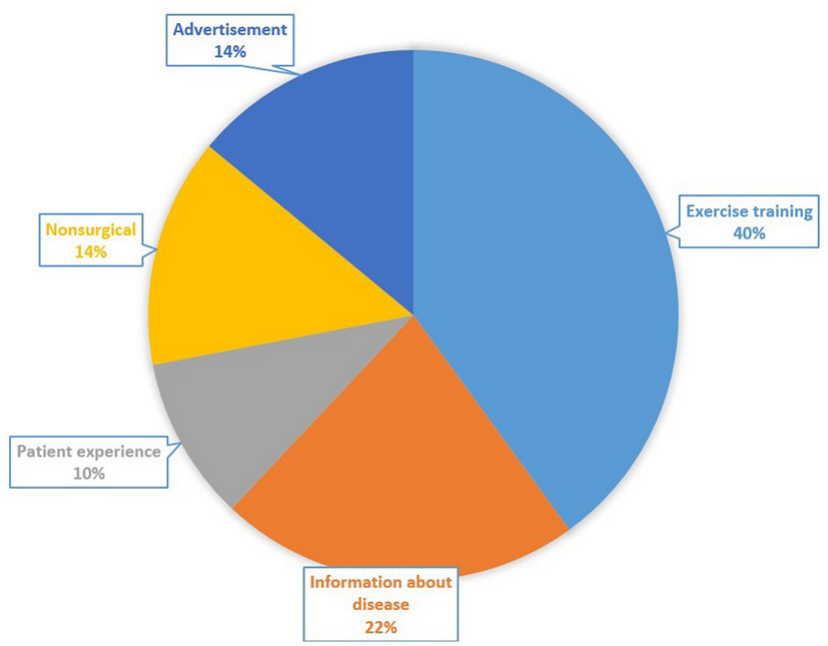
Categorical distribution of the videos based on content.

Among all contents, exercise training was the most frequently covered (20/50, 40%), followed by information about disease (11/50, 22%), non-surgical treatment (7/50, 14%), advertisement (7/50, 14%), and patient experience (5/50, 10%) (Figure 3).

### Information reliability and quality

The videos had a mean JAMA score of 2.56 (SD = 0.43), DISCERN of 2.55 (SD = 0.44), GQS of 2.86 (SD = 0.72), and NPSS of 2.90 (SD = 2.23). The reviewers had excellent agreement for the JAMA score (ICC, 0.909; 95% confidence interval, 0.846–0.947), DISCERN score (ICC, 0.923; 95% confidence interval, 0.858–0.958), GQS score (ICC, 0.927; 95% confidence interval, 0.876–0.958), and NPSS score (ICC, 0.939; 95% confidence interval, 0.895–0.965).

Additionally, between-group effects were calculated according to their upload source and contents. Significant differences between the uploading sources were observed for VPI (*P* = 0.012), JAMA (*P* < 0.0001), DISCERN (*P* < 0.0001), GQS (*P* = 0.001), and NPSS (*P* = 0.007), with videos from the academic group having the highest VPI, JAMA score, DISCERN, GQS, and NPSS. However, the VPI (*P* = 0.395), JAMA (*P* = 0.564), DISCERN (*P* = 0.869), GQS (*P* = 0.467), and NPSS (*P* = 0.329) did not statistically differ between the groups based on video content (Table 5).

**Table 5:** Mean quality and reliability scores per video source and video content variable.

**Grouping variable**	**VPI**	**JAMA**	**DISCERN**	**GQS**	**NPSS**
**Video content**					
Exercise training	482.83 ± 335.86	2.43 ± 0.31	2.49 ± 0.32	2.65 ± 0.48	2.20 ± 0.75
Information about disease	314.00 ± 352.61	2.75 ± 0.65	2.71 ± 0.72	3.09 ± 1.08	4.09 ± 3.82
Patient experience	874.36 ± 927.88	2.50 ± 0.35	2.56 ± 0.41	2.80 ± 0.75	3.20 ± 2.40
Non-surgical	943.62 ± 868.97	2.61 ± 0.18	2.51 ± 0.21	2.86 ± 0.35	2.43 ± 0.49
Advertisement	604.23 ± 684.87	2.64 ± 0.35	2.57 ± 0.27	3.00 ± 0.76	3.29 ± 1.58
*P*-value	0.395	0.564	0.869	0.467	0.329
**Video source**					
Academic	903.43 ± 745.28	3.06 ± 0.30	3.10 ± 0.40	3.50 ± 0.71	5.38 ± 4.09
Physician	631.82 ± 1071.73	2.75 ± 0.32	2.72 ± 0.24	3.20 ± 0.40	2.80 ± 0.40
Non-physician	709.11 ± 501.33	2.50 ± 0.24	2.52 ± 0.19	2.89 ± 0.45	2.16 ± 0.67
Medical	115.06 ± 99.02	2.58 ± 0.45	2.63 ± 0.45	3.00 ± 0.58	3.00 ± 1.53
Commercial	351.68 ± 208.23	2.39 ± 0.40	2.26 ± 0.32	2.43 ± 0.73	3.00 ± 1.77
Individuals	261.12 ± 182.32	1.95 ± 0.10	1.96 ± 0.37	1.80 ± 0.40	1.60 ± 0.49
Total	566.35 ± 613.49	2.56 ± 0.43	2.55 ± 0.44	2.86 ± 0.72	2.90 ± 2.23
*P*-value	0.012	< 0.001	< 0.001	0.001	0.007
The significant difference in *post-hoc* analysis^†^	Non-physician vs. medical	Academic vs. non-physician, academic vs. commercial, academic vs. individuals; Physician vs. individuals	Academic vs. non-physician, academic vs. commercial, academic vs. individuals	Academic vs. individuals; Physician vs. individuals; non-physician vs. individuals	Academic vs. individuals, academic vs. non-physician

Data are presented as mean ± SD.

† *Post-hoc* tests were performed using Bonferroni's method. GQS indicates Global Quality Score; JAMA, Journal of American Medical Association; VPI, video power index; NPSS, neck pain-specific score.

### Correlation analysis for factors influencing JAMA, DISCERN, GQS, and NPSS scores

In the total score correlation evaluation, the positive correlation between JAMA and discern was 84.3% (*p* < 0.0001), the positive correlation between JAMA and GQS was 87.3% (*p* < 0.0001), the positive correlation was 72.3% the percentage between JAMA and NPSS (*p* < 0.0001), the positive correlation between DISCERN and GQS was 84.3% (*p* < 0.0001), the positive correlation between DISCERN and NPSS was 72.0% (*p* < 0.0001), and the positive correlation between GQS and NPSs was 73.7% (*p* < 0.0001). However, there existed no significant correlation between the total VPI and the other four scores (Table 6).

**Table 6:** Spearman correlation analysis was applied to assess the correlation between JAMA, GQS, DISCERN, NPSS, and VPI.

		**VPI**	**JAMA**	**DISCERN**	**GQS**	**NPSS**
VPI	Correlation	1	0.152	0.143	0.149	−0.057
	*P*		0.293	0.321	0.303	0.629
JAMA	Correlation	0.152	1	0.843	0.873	0.723
	*P*	0.293		<0.001	<0.001	<0.001
DISCERN	Correlation	0.143	0.843	1	0.843	0.72
	*P*	0.321	<0.001		<0.001	<0.001
GQS	Correlation	0.149	0.873	0.843	1	0.737
	*P*	0.303	<0.001	<0.001		<0.001
NPSS	Correlation	−0.057	0.723	0.72	0.737	1
	*P*	0.629	<0.001	<0.001	<0.001	

JAMA, Journal of American Medical Association; GQS indicates Global Quality Score; NPSS, neck pain-specific score.

Multiple linear regression analysis suggested that a higher JAMA score was closely related with a higher probability of an academic (*P* < 0.0001), physician (*P* = 0.002), non-physician (*P* = 0.002) or medical (*P* = 0.002) upload source compared with an individual upload source. A higher DISECRN was closely related with a higher probability of an academic (*P* < 0.0001), physician (*P* = 0.005), non-physician (*P* = 0.006) or medical (*P* = 0.006) upload source than of an individual upload source. A higher GQS was closely related with academic (*P* < 0.0001), physician (*P* = 0.006), non-physician (*P* = 0.001) or medical (*P* = 0.003) upload sources than individuals upload sources. A higher NPSS was more associated with academic (*P* = 0.001) upload sources than with individual upload sources (Table 7).

**Table 7:** Multiple linear regression analysis of correlations between video characteristics and the JAMA score, DISCERN, GQS, and NPSS.

**Variable**	**Unstandardized beta (B)**	**95% CI**	**Standardized β**	***P*** **-value**
**JAMA score (*R*^2^ = 0.634)**				
Video source				
Academic	1.167	0.786–1.547	1.006	< 0.001
Physician	0.739	0.282–1.197	0.522	0.002
Non-physician	0.594	0.233–0.954	0.678	0.002
Medical	0.761	0.309–1.213	0.582	0.002
**DISCERN (*R*^2^ = 0.613)**				
Video source				
Academic	1.212	0.805–1.619	1.003	< 0.001
Physician	0.717	0.227–1.207	0.486	0.005
Non-physician	0.555	0.168–0.941	0.608	0.006
Medical	0.690	0.206–1.175	0.507	0.006
**GQS (*R*^2^ = 0.564)**				
Video source				
Academic	1.782	1.077–2.487	0.906	< 0.001
Physician	1.231	0.382–2.079	0.512	0.006
Non-physician	1.145	0.477–1.814	0.771	0.001
Medical	1.306	0.468–2.144	0.588	0.003
**NPSS (*R*^2^ = 0.500)**				
Video source				
Academic	4.393	2.060–6.726	0.722	0.001

JAMA, Journal of American Medical Association; GQS indicates Global Quality Score; NPSS, neck pain-specific score.

## Discussion

### Motivation and meaning of this study

Several factors piqued our interest in carrying out this work. The paramount reason is the ever-increasing number of patients coming to our outpatient clinic complaining of neck pain. Numerous studies have shown that neck pain is related to a lack of physical exercise (41, 42). Various restrictions during the epidemic have reduced activity and increased sedentary behavior (43), resulting in a significant increase in the number of neck pain patients. Second, many patients have conducted online research before visiting our clinic, and the information obtained sometimes contradicts the professional opinion of the doctor. With the internet penetration rate exceeding 50% worldwide (28), the internet and its online video repositories have increasingly been utilized as a source of health education for patients about a variety of medical conditions. However, the quality, reliability and scientific justification of the contained information remains unclear, given that the video content or uploaded source have not been assessed through peer review processes or minimum health-related standards. Thus, patients with neck pain may be exposed to inaccurate, incomplete even misleading information prior to seeking medical attention, making it quite necessary to be aware of the reliability and quality of education available to patients posted on YouTube. Since we orthopedic surgeons do not have the authority to review, edit or modify videos uploaded on YouTube or other sources, it is imperative that we at least understand how the Internet influences the patients concerning the commonly encountered diseases at the clinic.

Neck pain requires prompt diagnosis and treatment, which highlights the importance of accurate and comprehensive health education. In general, history and physical examination not only provide crucial clues as to whether the pain is neurologic or mechanical, but can also be used to identify “red flags” that may indicate serious pathology, such as cervical spondylotic myelopathy, atlantoaxial subluxation, and tumor metastasis. Magnetic resonance imaging is characterized by a high incidence of abnormalities in asymptomatic individuals, and should be considered in cases involving progressive neurologic deficits, in pain refractory to conventional treatment, and when referring patients for interventional treatment (37). For the treatment of neck pain, there are consistent weak or moderate intensity recommendations: advice and education, referral to exercise therapy/programs, oral analgesics and topical medications, plus psychotherapy, surgery, or multidisciplinary treatment for specific subgroups (11, 44, 45). Given the current epidemic, this is more important than ever, as patients with inadequate or inappropriate access to medical information about neck pain can miss out on individualized diagnosis and treatment for their condition, which can severely exacerbate or delay their conditions.

### Principal findings

To the best of our knowledge, there currently exist no studies evaluating the reliability and quality of YouTube videos providing medical information to patients with neck pain and identified factors associated with the overall video quality or reliability. According to our analysis, the reliability and quality of YouTube videos pertaining to neck pain were low, which is in consistent with the results of previous studies on YouTube videos within orthopedics. Erdem et al. (28) determined that the mean JAMA score (maximum of 4 points), GQS (maximum of 5 points), and kyphosis-specific score (maximum of 32 points) were 1.34, 1.68, and 3.02, respectively. MacLeod et al. (22) conducted YouTube-based research for Femoroacetabular Impingement information and found that the mean video quality assessment scores were 3.1 for diagnosis and 2.9 for treatment (maximum score of 16 points). Cassidy et al. (46) determined that the mean JAMA score and anterior cruciate ligament-specific score (maximum of 25 points) were 2.4 and 5.5, respectively. Hornung et al. (47) determined that the mean JAMA, GQS, and low back pain-specific score (maximum of 15 points) were 2.25, 2.29, and 3.83, respectively. Together, these data suggested that the reliability and quality of the YouTube videos on orthopedic disorders were low.

Even so, some studies conducted on YouTube found its reliability and quality to be high. First of all, our study is irrelevant to the subject of the above studies, this leads to different videos being retrieved on YouTube, which further leads to different details such as video upload sources. In the study conducted by Akyol Onder et al. (48) regarding dialysis, 32.6% (14/43) of videos were uploaded by universities/governments/professional societies. Ng et al. (49) searched on YouTube for information about lupus erythematosus and 49.7% (91/183) of videos were uploaded by professionals. Langford et al. (50) conducted the YouTube-based analysis for Spinal Cord Stimulation and 53.4% (55/103) were uploaded by hospitals, group practice or physician. However, in our study, only 16% (8/50) of videos were uploaded by professionals. Considering that professionals upload more high-quality videos (31, 51) and that most videos are uploaded by unqualified individuals and are not peer-reviewed, this may be the major reason why our study contradicted the minority of studies. In addition, in our study, particularly striking was the average NPSS of 2.90, with a maximum score of 18, highlighting the dilemma of the current video's lack of discussion of information specific to neck pain.

Once patients have researched their disease prior to their clinic visit, it is very challenging to properly inform and convince misguided patients to receive proper treatment and free them from prejudgment. Therefore, as clinicians, it is necessary for us to evaluate video quality and explore the factors that affect video quality to better guide patients on how to correctly find videos on the Internet that may be useful for their disease.

Despite the low reliability and educational quality of YouTube videos, those uploaded by academics scored significantly higher than those of other video sources. In addition, multiple linear regression analysis also supported that ownership (academic uploaders) is an important influencing factors that can be used to improve the reliability and quality of YouTube videos. This finding is consistent with those of other studies (31, 51). This may be explained by the fact that experts have more specialized knowledge, as well as the list of additional sources of information attached. When academic uploaders produce a video concerning neck pain, they should exert more effort to provide equalized information, list extra sources of information, and refer areas of uncertainty.

In contrast, the reliability and educational quality did not differ significantly among the video contents. The content of the videos included in the study mainly covered exercise training, information about disease, patient experience, and non-surgical and advertisement information. Most videos contain only 1 or 2 of the above categories. Particularly striking is the mean NPSS of 2.90, as the maximum possible score is 18 points, highlighting the dilemma that the current videos lack of discussion of neck pain-specific information. With the lack of availability of in-person medical information exchange during the COVID-19 pandemic, patients suffering from neck pain are at risk of insufficient or inadequate information if they decide to educate themselves regarding neck pain.

In our study, the popularity of videos was not significantly related to their quality or reliability, which was in agreement with previous research (27, 31). According to these findings, we can infer that the patients may find it challenging to differentiate between useful information and misleading information. Actually, of the 50 videos we included, some videos contained misleading information. These videos were often touted as “quick neck pain relief” when in fact they contained only simple neck exercises. This could cause some patients with severe diseases (e.g., spinal cervical spondylosis, neck trauma) to underestimate their condition, which could affect the diagnosis and treatment of the disease. This is more important than ever given the current situation, where insufficient or inappropriate access to medical information about neck pain can severely exacerbate or delay a patient's symptoms. Therefore, effective and prompt measures should be adopted to reduce inaccurate, incomplete (even harmful) information on YouTube platform.

### Challenges and solutions

Neck pain occupies one of the top five chronic pain conditions according to prevalence and years of disability, placing a considerable burden on individuals and the social economy. While most acute episodes resolve spontaneously, more than 30% of patients experience mild symptoms or relapse more than a year later, with genetic and psychosocial factors being persistent risk factors (11). To make matters worse, management of neck pain has been severely affected during the COVID-19 pandemic (18, 52–55). Given the explosive growth of the Internet as a fast and wide source of medical information in all areas of life, an increasing number of orthopedic patients have searched for medical information on YouTube, while the overall quality and reliability of relevant videos is poor. Most videos are uploaded by unqualified individuals and are not peer-reviewed, leading to the dissemination of inaccurate and low-quality data. Many studies have reported that a high percentage of videos posted on YouTube even provide misleading information (50, 51). The alarming state of YouTube platforms can easily lead to misinterpretation between patients and their doctors, hindering patients from proper selection and timely treatment, which may worsen their conditions.

A previous article recommended that a process of peer review during submission might be an possible solution (56), but the process not only requires experts and scholars to review but also consumes considerable time. Consistent with the conclusions of many previous studies (31, 51), our study demonstrated that YouTube videos uploaded from academics had a significantly higher quality and reliability than those posted by individuals. Hence, these professionals from academic groups should exhaust their expertise and provide patients with high-quality videos on YouTube as a source of information. Referring to the JAMA and DISCERN scoring scale, another feasible suggestion would be to ask video uploaders to provide their credentials or affiliations, state the references and sources for content and mention areas of uncertainty.

In addition, the perception and understanding of the video will depend on the user's level of knowledge of the subject. In our study, we aimed to assess the quality and reliability of the medical information shared on YouTube regarding neck pain with internationally recognized evaluation standards and to identify factors associated with the overall video quality or reliability. According to our results, YouTube videos pertaining to neck pain contain low quality, low reliability, and incomplete information. Even the videos from academics or physicians are inadequate in terms of quality and instructiveness. Therefore, there is a need not only to improve the background of the viewer's expertise or to make the expert's videos readable through health education, but most urgently to improve the quality and reliability of the videos and the accuracy and comprehensiveness of the information they contain. Professional associations or credible healthcare organizations should introduce educational videos on relevant diseases that will meet all JAMA criteria, have a GQS of 4 or higher, contain adequate, accurate, and comprehensive information about the patient presentation (symptoms and patients population), evaluation and diagnosis, and treatment alternatives of the disease, and about its possible results and complications, and will not negatively impact the clinician-patient relationship (28).

### Limitations

There are a few inevitable limitations in our study. First, the assessment scoring systems that we used (NPSS) are subjective and unvalidated. The NPSS included comprehensive contents of neck pain, while almost all YouTube videos focused on a specific topic and have a short running time of ~10 min or less. As such, it tended to be intractable to present all NPSS checklists. Nonetheless, considering that existing scoring criteria only non-specifically rate video quality rather than video content and no validated scales existed for assessing the content of video information, we designed quality assessment checklists based on a comprehensive review of relevant expert discussion and neck pain literature. Correlation analysis showed that NPSS was significantly correlated with JAMA, DISCERN, or GQS, which suggested that NPSS has a good application prospect. Second, we searched YouTube for the top 50 videos for “neck pain” by relevant. Actually, a default “relevance” sorting option was adopted by many studies when evaluating the reliability and quality of YouTube videos providing medical information to patients (22, 29, 32, 57–60). The default ranking option—“Relevance”—was the most commonly used option in the YouTube ranking algorithm (relevance, upload date, number of views, rating) (61, 62). This search strategy may miss some videos that have low views or hits but may be of high quality, but it is a practical way for casual users of YouTube to obtain information. Third, the characteristics of videos, such as the number of dislikes and comments, are constantly being updated. Thus, these video data are accurate only on the date of the search. Fourth, given that YouTube is the only online video archive that has been queried to evaluate educational content about neck pain, there is also the possibility of some selection bias. However, given that YouTube occupies the most influential and popular video hosting platform, we believe that using YouTube is an appropriate and clinically relevant platform as many patients access. Fifth, in retrieving the video, we did not use other synonyms for neck pain for the analysis. The reason for this was not only that we referred to the majority of the literature (27, 29, 63–65), but also such an analysis (use other synonyms) would divert our study from the intended course of evaluating the quality of information on a medical illness retrieved from YouTube and that the results returned upon searching with common keywords were mostly videos either not related to neck pain (28). Although our search strategy may miss some videos that were less “popular,” this strategy was the actual method by which casual YouTube users obtained information. Finally, we used the search item “neck pain” on YouTube and all videos were in English, making the conclusion cannot be applied to other languages.

## Conclusion

In particular, the current COVID-19 pandemic has highlighted the potential benefits of web-based education for patients suffering from chronic diseases. In light of its high popularity and ease to access, YouTube can be utilized to provide patients with timely medical information and tutorials for neck pain. However, this study suggested that videos concerning neck pain posted on YouTube showed low reliability and quality. The reliability or quality of the video is significantly related to the academic upload source. Video popularity was not associated with video reliability or quality, which suggests that popular videos do not guarantee good content quality. To ensure the dissemination of useful information, YouTube videos must be posted by academics to upload sources and strive to provide high-quality videos that aid in patient diagnosis and treatment.

## Data availability statement

The original contributions presented in the study are included in the article/Supplementary material, further inquiries can be directed to the corresponding author/s.

## Author contributions

XZ and HL participated in the conceptualization of the paper. XZ and YY conducted the data searches on the internet. Y-WS, K-RZ, and L-TM extracted relevant and analysis data. CD, B-YW, YM, and HL critically reviewed the manuscript for important intellectual content. XZ structured and wrote the paper. All authors read and approved the final manuscript.

## Funding

This study was supported by the National Natural Science Foundation of China (82172522), Sichuan Province Science and Technology Support Program of China (Nos. 2020YFS0089 and 2020YFS0077), Postdoctor Research Project, West China Hospital, Sichuan University (No. 2019HXBH063), and the Postdoctoral Science Foundation of China (No. 2020M673240).

## Conflict of interest

The authors declare that the research was conducted in the absence of any commercial or financial relationships that could be construed as a potential conflict of interest.

## Publisher's note

All claims expressed in this article are solely those of the authors and do not necessarily represent those of their affiliated organizations, or those of the publisher, the editors and the reviewers. Any product that may be evaluated in this article, or claim that may be made by its manufacturer, is not guaranteed or endorsed by the publisher.

## Supplementary material

Raw scores of JAMA score, DISCERN, and GQS are listed in Supplementary File 1.
